# Assessment of the Quality, Understandability, and Reliability of YouTube Videos as a Source of Information on Basal Cell Carcinoma: Web-Based Analysis

**DOI:** 10.2196/29581

**Published:** 2022-03-11

**Authors:** Theresa Steeb, Lydia Reinhardt, Matthias Harlaß, Markus Vincent Heppt, Friedegund Meier, Carola Berking

**Affiliations:** 1 Department of Dermatology Universitätsklinikum Erlangen Friedrich-Alexander-Universität Erlangen-Nürnberg Erlangen Germany; 2 Comprehensive Cancer Center Erlangen - Europäische Metropolregion Nürnberg Erlangen Germany; 3 Department of Dermatology University Hospital Carl Gustav Carus Technische Universität Dresden Dresden Germany

**Keywords:** basal cell carcinoma, YouTube, videos, patient education, shared decision-making, quality, reliability, internet, information

## Abstract

**Background:**

Patients with skin cancer increasingly watch online videos to acquire disease-related information. Until now, no scientific evaluation of the quality of videos available for German-speaking patients with basal cell carcinoma (BCC) has been performed.

**Objective:**

In this study, we aimed to identify and evaluate videos about BCC provided on YouTube.

**Methods:**

A video search on YouTube was conducted in July 2020, using German BCC-related keywords (eg, “Basalzellkarzinom,” “Basaliom,” “weißer hautkrebs,” and “heller hautkrebs”). The first three pages (ie, 60 videos) were searched by two independent researchers for each keyword. Two authors evaluated videos that met the predefined eligibility criteria. The quality of the information of the videos was evaluated using the DISCERN tool and the Global Quality Scale (GQS). The understandability and actionability were assessed with the Patient Education Materials Assessment Tool for Audiovisual Materials (PEMAT-A/V). The reliability was assessed with the JAMA (Journal of the American Medical Association) criteria score. Subgroup differences were identified using the Kruskal-Wallis test.

**Results:**

A total of 41 videos were included in the evaluation. The mean assessment scores were as follows: DISCERN, 3.3 (SD 0.80); GQS, 3.8 (SD 1.1); JAMA, 27.74% (SD 22.1%); understandability, 70.8% (SD 13.3%); and actionability, 45.9% (SD 43.7%). These values indicated that the videos were of medium to good quality and had good understandability, low actionability, and poor reliability. The quality of videos provided by health professionals was significantly higher than that of videos provided by laypersons.

**Conclusions:**

Optimization of health-related videos about BCC is desirable. In particular, adaptation to reliability criteria is necessary to support patient education and increase transparency.

## Introduction

Cutaneous basal cell carcinoma (BCC) represents the most common malignant tumor type in Central Europe, accounting for more than 80% of all epithelial skin carcinomas [1,2]. These tumors typically occur among fair-skinned individuals and are located most commonly on the head and neck, followed by the trunk and extremities [3]. The incidence of BCC continues to increase each year, with a current annual incidence of approximately 200 cases per 100,000 persons in Germany. However, the actual number is estimated to be much higher because cancer registries only document the first occurrence of BCC, and multiple tumors are not recorded [2,4]. BCC is rarely fatal, and surgical interventions remain to be the gold standard of treatment [1,5,6].

Patients with cancer in Germany commonly prefer to attend physician consultations in order to acquire disease-related information [7]. However, the physician’s time for a consultation is usually limited, while patients receive a large amount of medical and treatment-related information. Thus, patients may struggle with understanding all of the information provided and may subsequently feel inadequately informed [8]. While medical consultations and written information remain to be the most important sources of health information for patients, a steadily increasing number of patients are seeking health information on the internet [7,9-11]. YouTube is an open-access video-sharing platform, ranking second among the most-accessed websites worldwide, as it counts 5 billion visits per day and 1 billion hours watched daily [12]. It is increasingly used to disseminate health-related information and has become an easily accessible source for patients to acquire information related to their diseases [13]. The distribution of medical information to such a huge audience offers invaluable opportunities but also challenges, as the quality of unfiltered information posted can be of low scientific quality [14]. Information may even be misleading or harmful, as the credibility of the providers cannot be verified, and quality control of these videos has not yet been established [15-17]. Until now, no scientific evaluation of the quality of videos available for German-speaking patients with BCC has been performed. Therefore, the aim of this study was to identify YouTube videos about BCC and to assess their quality, reliability, usability, and understandability. The results of this study may encourage shared decision-making and be beneficial for both patients and health care providers in order to recommend appropriate videos to their patients.

## Methods

### Search Strategy

A video search on YouTube was conducted in July 2020, using German BCC-related keywords (eg, “Basalzellkarzinom,” “Basaliom,” “weißer hautkrebs,” and “heller hautkrebs”). The standard search options provided by YouTube were maintained. The first three pages (ie, 60 videos) were searched by two independent researchers for each keyword using Internet Explorer 11 (Microsoft). It has been observed that a significant proportion of users watch videos from only the first three pages. Furthermore, a similar methodology has been used in previous studies related to YouTube videos [18,19].

### Eligibility Criteria

To be eligible for evaluation, videos had to meet the following inclusion criteria: (1) contain information referring to BCC, (2) be accessible for free and for all users, and (3) provide information in the German language. Videos were excluded if they were commercials, they did not have sound, they presented only photos, or if the duration was less than one minute. All search results were screened for duplicates, and the predefined eligibility criteria were applied.

### Grouping of Videos

Due to the variety of the video providers, the videos were grouped according to their original source into the following categories: layperson, health professional (ie, hospital or practice), educational provider, noncommercial provider or professional society, pharmaceutical company, health portal, and unclassified. For television or news reports, we distinguished whether they were uploaded by the official channel or reuploaded by private providers.

### Data Management

The available baseline information (ie, URL, title, name of the provider, video length, and year of upload) of each selected video was documented. Additionally, the numbers of views, likes, and dislikes were extracted. With this information, we calculated the video power index (VPI) to assess the popularity of the videos. The VPI is calculated as follows:

VPI = number of likes / (number of likes + number of dislikes) × 100

The baseline information was extracted to an internally piloted data extraction sheet using Microsoft Excel 2010.

Two reviewers (TS and MH) independently assessed the videos’ quality of information, reliability, and understandability. Prior to the assessment, the use of the assessment tools was piloted by independently evaluating the first five videos to discuss potential difficulties and resolve questions.

### Quality of Information

The DISCERN tool is commonly used to assess the quality of cancer information and was developed for laypersons [20]. A modified German version of this tool was used in this study, consisting of nine items that were used (1) to review a video’s transparency (items 1-6), (2) to review a video’s content (items 7 and 8), and (3) to give an intuitive assessment summary (item 9). Items were scored on a 5-point scale ranging from 1 (“criterion is not met at all”) to 5 (“criterion is fully met”; Multimedia Appendix 1). Thus, videos that were rated, on average, 4 or higher were considered to be of good quality, those rated from 2 to below 4 were considered medium quality, and those rated less than 2 were considered low quality. A maximum of 45 points could be achieved.

Additionally, the Global Quality Scale (GQS) was used. The GQS includes a 5-point scale ranging from 1 (“low quality”) to 5 (“high quality”) [21]. Videos scoring 4 or 5 points were rated as high quality, those scoring 3 points were rated as medium quality, and those scoring 1 or 2 points were rated as low quality.

### Understandability and Actionability

The Patient Education Materials Assessment Tool for Audiovisual Materials (PEMAT-A/V) was chosen to assess the individual videos’ understandability and actionability. The understandability section comprises 13 items that covered content, word choice and style, organization, layout and design, and the use of visual aids [22]. The second section covers actionability by four items. Each item can be scored as 0 (“disagree”), 1 (“agree”), or N/A (“not applicable”). Then, percentage scores for both sections are calculated by dividing the number of achieved points by the number of items the video was evaluated on in each section. PEMAT-A/V scores range from 0% to 100%, with higher values generally indicating better understandability or actionability.

### Accuracy, Utility, and Reliability

The accuracy, utility, and reliability of each video source were explored according to the JAMA (Journal of the American Medical Association) benchmark criteria [23]. These four criteria included authorship (ie, authors, contributors, affiliations, and credentials), attribution (ie, references and sources used for the content and copyright information), disclosures (ie, sponsorship, advertising, commercial funding, and potential conflicts of interest), and currency (ie, dates of posted and updated information). Each item can be scored as 0 (“disagree”) or 1 (“agree”). Next, we calculated percentages of fulfilled items. The higher the value, the more accuracy, utility, and reliability elements were fulfilled.

### Harms and Benefits

In order to summarize their potential benefit or harm, the videos were rated on an adapted 3-point scale as to whether they were perceived to be useful, neutral, or harmful for potential audiences [24]. Useful videos were judged to contain correct information and to be of value to patients, whereas harmful videos contained misleading or false information.

### Statistical Analysis

Statistical analyses were conducted using SPSS Statistics for Windows (version 24; IBM Corp). Descriptive analyses included mean (SD) or median (range). Subgroup differences were explored using the Kruskal-Wallis test. The relationship between the individual items of the tests was examined using Spearman correlation. Statistical significance was set at *P*≤.05. The interrater agreement of the two reviewers was determined using the intraclass correlation coefficient, as well as by determining the interitem correlation, *r*, between the individual reviewers.

## Results

### Video Identification and Baseline Characteristics

**Figure 1:**
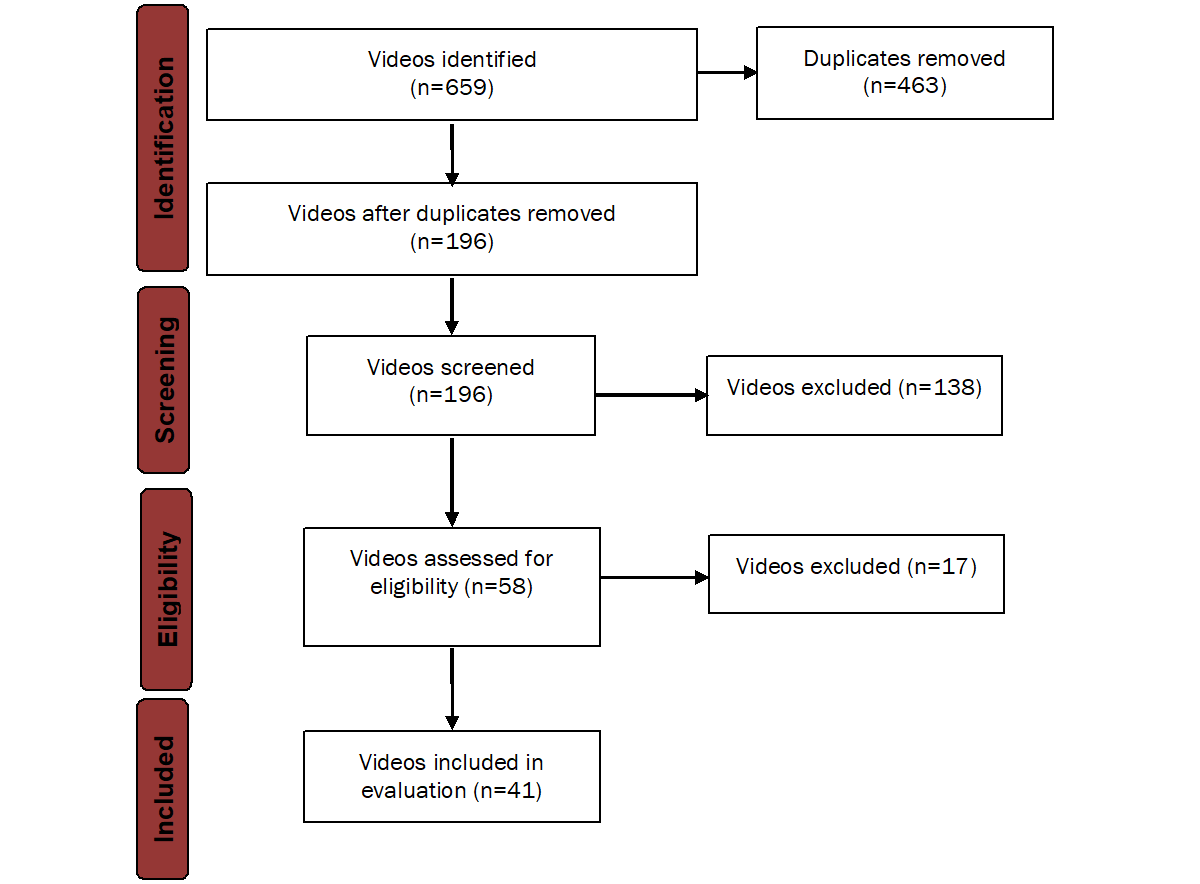
Flowchart showing the identification process of the videos.

Our search identified 659 videos. Following a multistep process, three review authors (TS, MH, and LR) screened the videos for duplicates and checked them for compliance with the predefined eligibility criteria. Finally, 41 individual videos were considered for assessment (Figure 1). Most videos were provided by health professionals (15/41, 37%), followed by laypersons (6/41, 15%) and health portals (6/41, 15%). Furthermore, 10% of the videos (4/41) were offered by educational providers, and 7% (3/41) of the videos were TV reports uploaded by official TV channels or reuploaded by private providers. Out of 41 videos, 2 (5%) providers remained unclear.

The videos were uploaded between 2011 and 2020, with the majority (30/41, 73%) uploaded after 2017 (Table 1 and Multimedia Appendix 2). The number of views ranged from 25 to 386,195, with a mean of 27,853 views. The video length (minutes: seconds) ranged from 1:04 to 91:36. In 78% (32/41) of the videos, the duration was less than 10 minutes. The number of likes ranged from 0 to 17,925, with a median of 22. Most likes were given on a video dealing with the personal BCC history of a German influencer (video #20). The number of dislikes ranged from 0 to 333. The VPI was evaluable for 33 videos and ranged from 40 to 100.

Overall, video #8 (“Hautkrebs - Ein Überblick über Typen und Therapien”; Multimedia Appendix 2), provided by health professionals, and video #35 (“Weißer Hautkrebs – ein Patienteninformationsfilm”), created by a professional society, were rated best among all videos. Both videos gave an overview on the disease course. In contrast, video #30 (“Verjüngung mit Uta Baranovskyy: Weißer Hautkrebs Teil 3”) provided by a layperson was rated the worst due to misleading information regarding the treatment of BCC. 

**Table 1:** Overview of baseline characteristics, quality, understandability, actionability, and reliability of the videos according to the respective categorization of the provider.

Characteristic	Provider								
	All	Layperson	TV reupload by private account	Health prof.^a^	Official TV report	Educ.^b^ provider	Prof. society or NC^c^ provider	Health portal	Unclear
Videos, n (%)	41 (100)	6 (15)	3 (7)	15 (37)	3 (7)	4 (10)	2 (5)	6 (15)	2 (4.9)
Views, mean (SD)	27,853 (70,693)	101,427 (1,591,378)	46,049 (77,209)	4221 (3105)	68,499 (57,295)	2177 (2751)	37,602 (25,232)	6980 (6836)	326 (350)
Video length (min:s), mean (range)	11:12 (1:04- 91:36)	15:07 (1:15- 37:44)	6:31 (4:20- 9:12)	8:25 (1:16- 65:50)	12:41 (4:04- 29:13)	5:30 (1:04- 16:52)	25:51 (8:42- 43.01)	2:48 (1:49- 3:57)	46:55 (2:15- 91:36)
Year of upload, range	2011- 2020	2017- 2019	2011- 2018	2012- 2020	2012- 2020	2016- 2019	2016	2013- 2020	2018- 2020
Likes, mean (range)	672 (0-17,925)	4383 (20-17,925)	54 (0-139)	19 (0-59)	172 (53-344)	14 (0-42)	24.50 (0-49)	26 (0-68)	3 (2-3)
Dislikes, mean (range)	15 (0-333)	74 (0-333)	12 (0-34)	1 (0-3)	28 (12-51)	1 (0-3)	5 (0-9)	2 (0-5)	0 (0)
DISCERN score^d^, mean (SD)	3.30 (0.80)	1.95 (0.41)	3.50 (0.44)	3.64 (0.53)	3.08 (0.94)	3.33 (0.41)	3.72 (0.93)	3.46 (0.70)	4.03 (0.04)
GQS score^e^, mean (SD)	3.76 (1.13)	1.83 (0.41)	4.12 (0.29)	4.47 (0.64)	3.33 (1.26)	3.50 (0.41)	4.00 (0.71)	3.83 (1.21)	4.25 (1.06)
PEMAT-A/V score^f^ (% U), mean (SD)	70.84 (13.32)	60.28 (11.50)	76.77 (10.16)	75.32 (13.26)	78.59 (11.33)	71.44 (11.49)	74.66 (18.16)	61.55 (10.92)	71.14 (19.61)
PEMAT-A/V score^f^ (% A), mean (SD)	45.94 (43.74)	55.56 (50.18)	27.78 (25.46)	56.67 (40.21)	33.33 (33.33)	25.00 (50.00)	50.00 (70.71)	36.11 (37.14)	50.00 (70.71)
JAMA score^g^ (%), mean (SD)	27.74 (22.10)	12.50 (15.81)	20.83 (7.22)	30.00 (16.23)	37.50 (21.65)	43.75 (38.86)	62.50 (17.67)	12.50 (15.81)	31.25 (26.52)

^a^prof: professional.

^b^educ: educational.

^c^NC: noncommercial.

^d^DISCERN items were scored on a 5-point scale ranging from 1 (“criterion is not met at all”) to 5 (“criterion is fully met”); videos were considered good quality (≥4), medium quality (≥2 to <4), or low quality (<2).

^e^GQS: Global Quality Scale; the GSQ was scored a 5-point scale ranging from 1 (“low quality”) to 5 (“high quality”); videos were considered high quality (4 or 5), medium quality (3), or low quality (1 or 2).

^f^PEMAT-A/V: Patient Education Materials Assessment Tool for Audiovisual Materials; scores range from 0% to 100%, with higher values indicating better understandability (U) or actionability (A).

^g^JAMA: Journal of the American Medical Association; each of four criteria were scored as 0 (“disagree”) or 1 (“agree”); scores range from 0% to 100%, with higher values indicating higher reliability.

### Quality: DISCERN and GQS Results

Out of 45 points in total, the 41 individual videos ranged between 10.5 and 35.0 points according to the DISCERN tool. The mean DISCERN scores per video ranged from 1.31 to 4.38 points, with an average mean score of 3.31 (SD 0.80) points, indicating medium quality (Table 1). Most score deductions were due to lacking information about the sources used to create the respective video or missing complementary information. The mean GQS score was 3.8 (SD 1.1) points, indicating medium quality as well.

### Understandability and Actionability: PEMAT-A/V Results

The average PEMAT-A/V score was 70.84% (SD 13.32%, range 43.18%-100%) for understandability and 45.94% (SD 43.74%, range 0%-100%) for actionability. Most score deductions for the understandability domain were due to a lack of a summary and because no visual aids were deployed. For the actionability domain, information was often missing regarding the interpretation of certain figures in order to take action.

### Accuracy, Utility, and Reliability: JAMA Results

In total, a mean of 27.74% (SD 22.1%, range 0%-87.5%) of the JAMA benchmark criteria were fulfilled, indicating rather poor reliability. The main reasons for score deductions were missing information regarding the currency of videos (ie, the upload date) and missing disclosure of the provider.

### Harms and Benefits

A total of 49% (20/41) of the videos were evaluated as useful, 7% (3/41) were evaluated as harmful, and the remaining videos were evaluated as neither beneficial nor harmful. All videos estimated to be harmful were provided by laypersons.

### Interrater Agreement

We calculated intraclass correlation coefficients ranging from 0.940 to 0.955 with a Cronbach α of .973, indicating high overall interrater agreement concerning the assessments by the DISCERN tool, the GQS, the JAMA criteria, and the PEMAT-A/V. The interitem correlation, *r*, was 0.949, indicating high individual agreement among the two reviewers when assessing the individual items.

### Subgroup Analyses

Significant differences in video quality, according to the DISCERN tool and the GQS, were identified between videos provided by laypersons and health professionals (*P*=.01; ie, videos by health professionals were judged as having higher quality than those provided by laypersons).

Regarding the assessment of whether videos were beneficial or not, differences were found in terms of the quality of the videos. Videos rated as beneficial showed significantly better quality in comparison to those rated as harmful (DISCERN: *P*=.004; GQS: *P*=.002) and neutral (DISCERN: *P*=.006; GQS: *P*<.001), according to the DISCERN tool and the GQS. No further subgroup differences were identified.

### Correlation Analysis

A significant positive correlation was found between DISCERN and GQS values (*r*=0.836) as well as between DISCERN values and reliability and understandability criteria (*r*=0.488 and *r*=0.460, respectively; Table 2). In addition, the quality according to the GQS also significantly correlated with the reliability (*r*=0.426) and understandability (*r*=0.482) of the videos. Furthermore, the longer the duration of a video, the more understandability (*r*=0.454) and actionability (*r*=0.314) items had been deployed. No further significant correlations between the baseline characteristics and the quality, reliability, understandability, or actionability of the videos were identified.

**Table 2:** Correlation analysis (Spearman *r* and two-tailed *P* value) among the research variables.

Variable			Baseline characteristics						Quality			Reliability		PEMAT-A/V^a^	
			Likes	Dislikes	Views	Duration	Uploads	DISCERN		GQS^b^	JAMA^c^		Understandability		Actionability
**Baseline characteristics**															
	**Likes**														
		*r*	1	0.784^d^	0.604^d^	0.375^e^	–0.060	–0.085		–0.082	–0.084		0.031		0.215
		*P* value	—^f^	<.001	<.001	.02	.71	.60		.61	.60		.85		.18
	**Dislikes**														
		*r*	0.784^d^	1	0.619^d^	0.228	–0.323^e^	–0.186		–0.151	0.037		–0.119		0.066
		*P* value	<.001	—	<.001	.15	.04	.25		.35	.82		.46		.68
	**Views**														
		*r*	0.604^d^	0.619^d^	1	0.200	–0.380^e^	0.086		0.003	0.068		0.029		0.055
		*P* value	<.001	<.001	—	.21	.01	.59		.99	.67		.86		.74
	**Duration**														
		*r*	0.375^e^	0.228	0.200	1	0.226	–0.013		–0.025	0.115		0.454^d^		0.314
		*P* value	.02	.15	.21	—	.16	.93		.87	.47		<.001		.06
	**Uploads**														
		*r*	–0.060	–0.323^e^	–0.380^e^	0.226	1	0.059		0.106	–0.152		0.300		0.157
		*P* value	.71	.04	.01	.16	—	.72		.51	.34		.06		.33
**Quality measures**															
	**DISCERN**														
		*r*	–0.085	–0.186	0.086	–0.013	0.059	1		0.836^d^	0.488^d^		0.460^d^		0.135
		*P* value	.60	.25	.59	.93	.72	—		<.001	<.001		<.001		.40
	**GQS**														
		*r*	–0.082	–0.151	0.003	–0.025	0.106	0.836^d^		1	0.426^d^		0.482^d^		0.186
		*P* value	.61	.35	.99	.87	.51	<.001		—	<.001		<.001		.24
**Reliability measures**															
	**JAMA**														
		*r*	–0.084	0.037	0.068	0.115	–0.152	0.488^d^		0.426^d^	1		0.469^d^		–0.052
		*P* value	.60	.82	.67	.47	.34	<.001		<.001	—		<.001		.75
**PEMAT-A/V measures**															
	**Understandability**														
		*r*	0.031	–0.119	0.029	0.454^d^	0.300	0.460^d^		0.482^d^	0.469^d^		1		0.220
		*P* value	.85	.46	.86	<.001	.06	<.001		<.001	<.001		—		.17
	**Actionability**														
		*r*	0.215	0.066	0.055	0.314^e^	0.157	0.135		0.186	–0.052		0.220		1
		*P* value	.18	.68	.74	.05	.33	.40		.24	.75		.17		—

^a^PEMAT-A/V: Patient Education Materials Assessment Tool for Audiovisual Materials.

^b^GQS: Global Quality Scale.

^c^JAMA: Journal of the American Medical Association.

^d^The correlation is significant at a significance level of <.001 (two-tailed).

^e^The correlation is significant at a significance level of .05 (two-tailed).

^f^Not applicable.

## Discussion

### Principal Findings

In this study, 41 YouTube videos about BCC have been systematically identified and evaluated by two independent reviewers. For the first time, we present an in-depth and objective assessment of the quality, understandability, and reliability of the information about BCC provided by YouTube videos on this subject. There were more than 1 million views among the 41 videos identified in our search, highlighting the importance of the internet and platforms like YouTube as sources of health information. Half of the assessed videos were estimated to be beneficial for patients, showing that YouTube may be an important tool for information broadcasting. The percentage of beneficial videos was similar compared to the results of previous studies evaluating video contents about other diseases [25-27].

Our results complement the currently available evidence on informational material available for other types of skin cancer, such as videos, brochures, or websites [14,27,28]. Our evaluation shows that currently available BCC videos were, overall, of medium to good quality and understandability but had low actionability and poor reliability. In addition, we have shown that videos of longer duration applied more understandability and actionability items and that the quality of videos provided by health professionals was significantly higher than that of videos provided by laypersons.

Interestingly, none of the videos identified in our search were provided by pharmaceutical companies, which sharply contrasts with our previous search and evaluation of videos on melanoma [27]. In that study, 16% of the videos had been created by pharmaceutical companies and nearly one-third by laypersons, while most videos on BCC had been supplied by health professionals. A potential explanation might be that pharmaceutical companies offer more videos on melanoma, as the interest in disease-specific knowledge is judged to be more important due to the complexity and abundance of different therapy regimens. Nevertheless, our evaluation revealed that videos about BCC provided by health professionals scored the best ratings in terms of quality, understandability, and reliability. This may be explained by the fact that these providers have better resources and scientific backgrounds to produce such high-quality videos.

In summary, the quality, understandability, and reliability of the BCC videos were comparable to those about melanoma [27]. However, BCC videos were judged to score more points on actionability items and fewer points on reliability items. Notably, the most likes were awarded for the two videos uploaded by a female influencer describing her own personal history with BCC as well as her therapy and follow-up. While these videos were mostly inferior in comparison to other videos, they highlight that the involvement of testimonials or influencers might be a feasible approach to maximize the awareness of skin cancer, in general, and to promote preventive measures. However, on the other hand, they may also use their coverage to distribute incorrect or harmful information.

YouTube is a growing online video platform providing easy access [12] with steadily increasing popularity among patients and medical professionals [29]. Distribution of medical information to such a huge audience offers invaluable opportunities but also risks of misinformation and biased presentation. Since the accuracy of online information is variable and since there is no peer review of such videos, the credibility and trustworthiness of the providers cannot be verified [15-17]. Moreover, quality certificates, like HONcode (Health on the Net Foundation Code of Conduct), which are awarded for reliable health-related webpages, are missing for YouTube videos [30]. Additionally, YouTube can be used as an advertising tool. As users can share their personal opinions without sufficient information and experience, videos may mislead patients and affect the physician-patient relationship [31]. Obtaining correct information from reliable sources is crucial, as it increases patients’ satisfaction and empowerment and may improve treatment results [32,33]. Efforts should be undertaken to introduce regular quality control of videos with medical content on YouTube.

We are aware that this study has some limitations. YouTube search results are highly dynamic and will change when new videos are uploaded and when old videos are removed. Additionally, we did not include videos with restricted access (eg, asking for log-in information).

### Conclusions

Overall, our study demonstrates that online videos on BCC are currently of medium to good quality and are predominantly uploaded by health professionals. However, the reliability of the videos was poor. As more and more patients use online material, including YouTube videos, for acquiring disease-specific knowledge, it is crucial to ensure good quality, understandability, and reliability prior to publication. Thus, optimization of the videos is desirable. In particular, adaptation to reliability criteria is necessary to support patient education and increase transparency. Patients should be advised to check the sources of the videos and whether their content is up to date.

## Appendix

Multimedia Appendix 1 Overview of the different assessment tools used for the evaluation of the videos.

Multimedia Appendix 2 Summary of information about 41 videos on melanoma in German analyzed in this study.

## Abbreviations

BCC: basal cell carcinoma
GQS: Global Quality Scale
HONcode: Health on the Net Foundation Code of Conduct
JAMA: Journal of the American Medical Association
N/A: not applicable
PEMAT-A/V: Patient Education Materials Assessment Tool for Audiovisual Materials
VPI: video power index
