# Supplementary material for: Assessment of the Quality, Understandability, and Reliability of YouTube Videos as a Source of Information on Basal Cell Carcinoma: Web-Based Analysis
Source: JMIR Cancer. 2022 Mar 11;8(1):e29581. doi: 10.2196/29581 (PMC8956995; doi:10.2196/29581)
Supplement: Multimedia Appendix 1 [file cancer_v8i1e29581_app1.docx]

**Multimedia Appendix 1: Overview of the different assessment tools used for the evaluation of the videos.**

| **Category assessed** | **Tool** | **Score range** | **Interpretation** |
| --- | --- | --- | --- |
| Quality | DISCERN (modified version)  9 items covering   - a publication’s transparency (items 1-6) - content (items 7-8) - to give an intuitive assessment summary (item 9) | 1-45 | Higher values indicate higher quality |
|  | Global Quality Scale   - Poor quality, poor flow of the video, most information missing, not at all useful for patients - Generally poor quality and poor flow, some information listed but many important topics missing, of very limited use to patients - Moderate quality, suboptimal flow, some important information is adequately discussed but others poorly discussed, somewhat useful for patients - Good quality and generally good flow, most of the relevant information is listed, but some topics not covered, useful for patients - Excellent quality and excellent flow, very useful for patients | 1-5 | Higher values indicate higher quality |
| Reliability and accuracy | JAMA   - authorship (authors, contributors, affiliations, and credentials) - attribution (references and sources used for the content and copyright information) - disclosures (sponsorship, advertising, commercial funding, and potential conflicts of interests) - currency (dates of posted and updated information) | 0-100% | Higher percentage indicate higher reliability |
| Understandability and actionability | PEMAT-A/V   - Understandability: 13 items that cover content, word choice and style, organization, layout and design, and the use of visual aids - Actionability: 4 items | 0-100% | Higher percentage indicate higher understandability and actionability |
