# Supplementary material for: Assessment of the Quality, Understandability, and Reliability of YouTube Videos as a Source of Information on Basal Cell Carcinoma: Web-Based Analysis
Source: JMIR Cancer. 2022 Mar 11;8(1):e29581. doi: 10.2196/29581 (PMC8956995; doi:10.2196/29581)
Supplement: Multimedia Appendix 2 [file cancer_v8i1e29581_app2.docx]

**Multimedia Appendix 2: Summary of mean information about 41 videos on basal cell carcinoma in German analyzed in this study.**

| **#** | **Video Title** | **Provider** | **DISCERN** | **GQS** | **Under-standability** | **Actionability** | **JAMA** | **Usefulness** | **Duration (minutes)** | **VPI** | **Year** |
| --- | --- | --- | --- | --- | --- | --- | --- | --- | --- | --- | --- |
| 1 | #aproposgesund Thema: Wie gefährlich ist Hautkrebs? | health professional | 3.5 | 5 | 83.33 | 33.33 | 25 | useful | 06:22 | 98.33 | 2020 |
| 2 | BASALIOM an der NASE: OP Bilder und Ergebnis nach der Wundheilung einer Hautlappenplastik | health professional | 3.88 | 5 | 90.1 | 100 | 25 | useful | 06:26 | 94.44 | 2020 |
| 3 | Basaliom. Basalzellkarzinom: die wichtigsten Antworten auf Ihre Fragen | health professional | 4.25 | 5 | 87.5 | 100 | 25 | useful | 07:13 | 100 | 2020 |
| 4 | Behandlungsmöglichkeiten bei Hautkrebs | health professional | 3.81 | 4.5 | 77.27 | 100 | 37.5 | useful | 02:49 | 88.46 | 2012 |
| 5 | BLK Gesundheitsmagazin weißer Hautkrebs Operation Krankenhaus Weissenfels | Official TV report | 3.63 | 3.5 | 72.27 | 66.67 | 62.5 | neutral | 04:48 | 85.51 | 2013 |
| 6 | Eigentherapie von Gesichtsbasaliomen mit Schwarzer Salbe. Beginn Juni 2017. Dokumentation. Teil 4/6 | layperson | 2.38 | 2.5 | 71.67 | 0 | 37.5 | harmful | 37:44:00 | 83.33 | 2017 |
| 7 | Endlich Hoffnung für Patienten mit fortgeschrittenem weißen Hautkrebs | health portal | 3.13 | 3 | 72.27 | 0 | 25 | neutral | 02:34 | n.a. | 2013 |
| 8 | Forum (Live-Stream): Hautkrebs - Ein Überblick über Typen und Therapien | health professional | 4.19 | 5 | 100 | 100 | 62.5 | useful | 65:50:00 | 100 | 2020 |
| 9 | Fragen Sie Dr. Busch, 24. Folge: Wie gefährlich ist heller Hautkrebs? | health portal | 4.19 | 5 | 72.5 | 33.33 | 0 | useful | 03:57 | 94.12 | 2017 |
| 10 | Früherkennung: So schützen Sie sich vor Hautkrebs | Official TV report | 3.63 | 4.5 | 71.82 | 0 | 25 | neutral | 04:04 | 87.09 | 2012 |
| 11 | Gesundheit im Gespräch - Weißer und schwarzer Hautkrebs | unclear | 4.06 | 5 | 85 | 100 | 50 | useful | 91:36:00 | 100 | 2018 |
| 12 | Gibt es Risikofaktoren für den weissen Hautkrebs? | health professional | 3.44 | 5 | 62.27 | 100 | 25 | useful | 01:44 | n.a. | 2018 |
| 13 | Hautkrebs | education | 3.13 | 3 | 83.33 | 100 | 0 | neutral | 16:52 | 100 | 2019 |
| 14 | Hautkrebs - Talk Video - Basalzellkarzinom | layperson | 2.25 | 2 | 57.27 | 100 | 25 | neutral | 01:15 | 97.18 | 2017 |
| 15 | Hauttumoren - Entfernung und anschließende Wundversorgung | education | 3.94 | 4 | 77.65 | 0 | 87.5 | useful | 01:05 | 100 | 2017 |
| 16 | Hellen Hautkrebs erkennen und behandeln | TV report re-uploaded by private channel | 3.5 | 4 | 76.36 | 33.33 | 12.5 | useful | 04:20 | 95.83 | 2018 |
| 17 | Heller Hautkrebs - die Haut vergißt nichts | professional society/non-commercial provider | 3.06 | 3.5 | 61.82 | 0 | 50 | useful | 08:42 | 84.48 | 2016 |
| 18 | Heller Hautkrebs: Arten, Symptome, Photodynamische Therapie. Prof. Dr. Dirschka | health professional | 4.13 | 5 | 76.36 | 0 | 50 | useful | 03:24 | 90 | 2019 |
| 19 | Heller und Schwarzer Hautkrebs \| Dr. Uwe Schwichtenberg | unclear | 4 | 3.5 | 57.27 | 0 | 12.5 | neutral | 02:15 | 100 | 2020 |
| 20 | ICH HABE HAUTKREBS… | layperson | 1.63 | 1.5 | 47.73 | 100 | 0 | neutral | 09:39 | 98.18 | 2019 |
| 21 | MEDI-LOGIN - Dermatoskopie-Kurs Probelektion 7 - Basaliom | education | 3.13 | 3.5 | 67.5 | 0 | 25 | neutral | 01:04 | n.a. | 2019 |
| 22 | Meine Heilung vom weißen Hautkrebs | layperson | 1.94 | 1.5 | 56.82 | 100 | 0 | harmful | 10:50 | 93.42 | 2017 |
| 23 | OCT Basaliom | health professional | 2.13 | 3 | 61.82 | 0 | 0 | neutral | 01:16 | 87.5 | 2015 |
| 24 | OP Sklerodermiformes BASALIOM an der Nasenspitze. Entfernung + Lappenplastik + Hautverpflanzung | health professional | 3.75 | 4 | 81.36 | 100 | 25 | useful | 05:13 | 89.47 | 2020 |
| 25 | Operation eines Basalioms - Mohs' Surgery | health professional | 3.75 | 4.5 | 83.33 | 0 | 50 | useful | 05:34 | 100 | 2020 |
| 26 | Photodynamische Therapie | health portal | 2.5 | 3.5 | 56.82 | 100 | 0 | useful | 01:49 | n.a. | 2014 |
| 27 | Prof. Dr. Dirschka Basalzellkarzinom / Basaliom / heller Hautkrebs Centroderm | health professional | 4 | 5 | 67.27 | 100 | 37.5 | useful | 04:02 | 95.45 | 2019 |
| 28 | SO SEHE ICH JETZT AUS - Nach der Hautkrebs OP! | layperson | 2.19 | 2 | 76.82 | 33.33 | 12.5 | neutral | 13:00 | 98.89 | 2019 |
| 29 | Uniklinik Köln \| Augenklinik: Basaliom - Basalzellkarzinom (Patienteninformation) | health professional | 3.5 | 4.5 | 71.36 | 50 | 37.5 | neutral | 03:41 | 88 | 2017 |
| 30 | Verjüngung mit Uta Baranovskyy: Weißer Hautkrebs Teil 3 | layperson | 1.31 | 1.5 | 51.36 | 0 | 0 | harmful | 18:18 | 100 | 2018 |
| 31 | Was bedeutet Basaliom / weißer Hautkrebs? | health portal | 3.88 | 5 | 62.27 | 33.33 | 12.5 | useful | 02:16 | 95 | 2018 |
| 32 | Was ist ein knotiges Basalzellkarzinom | health portal | 2.94 | 2 | 43.18 | 50 | 0 | neutral | 03:11 | 100 | 2020 |
| 33 | Was ist weisser Hautkrebs? | health professional | 3.81 | 4 | 56.82 | 0 | 12.5 | useful | 01:28 | n.a. | 2018 |
| 34 | Was sind Basaliome und was kann man dagegen tun? Dr. Thorsten Wegner | health portal | 4.13 | 4.5 | 62.27 | 0 | 37.5 | neutral | 03:06 | 93.15 | 2014 |
| 35 | Weißer Hautkrebs - ein Patienteninformationsfilm | professional society/non-commercial provider | 4.38 | 4.5 | 87.5 | 100 | 75 | useful | 43:01:00 | n.a. | 2016 |
| 36 | Weißer Hautkrebs (Basalzellkarzinom) - Erkennen und behandeln | TV report re-uploaded by private channel | 3.94 | 4.5 | 66.82 | 50 | 25 | neutral | 09:12 | 80.35 | 2011 |
| 37 | Weißer Hautkrebs: Die Schattenseite der Sonne | TV report re-uploaded by private channel | 3.06 | 4 | 87.12 | 0 | 25 | neutral | 06:01 | n.a. | 2018 |
| 38 | Welche Arten von Hautkrebs gibt es? - MINI MED Talk mit Univ.-prof. Dr. Franz Trautinger | education | 3.13 | 3.5 | 57.27 | 0 | 62.5 | neutral | 03:01 | 40 | 2016 |
| 39 | Wie Hautkrebs entsteht und wie er behandelt werden kann | health professional | 3.44 | 4 | 77.5 | 66.67 | 25 | useful | 09:04 | 85.71 | 2018 |
| 40 | Wie wird Hautkrebs behandelt? | health professional | 3 | 3.5 | 52.73 | 0 | 12.5 | neutral | 02:15 | n.a. | 2018 |
| 41 | Hautkrebs-Experten beantworten eure Fragen \| BILD Live vom 14.02.2020 | Official TV report | 2 | 2 | 91.67 | 33.33 | 25 | neutral | 29:13:00 | 81.54 | 2020 |

**Abbreviations:** GQS = global quality scale; JAMA = Journal of American Medical Association (JAMA) score; n.a. = not available; PEMAT = Patient Education Materials Assessment Tool; VPI = video power index
